# NRF2 is required for structural and metabolic maturation of human induced pluripotent stem cell-derived ardiomyocytes

**DOI:** 10.1186/s13287-021-02264-2

**Published:** 2021-03-24

**Authors:** Xinyuan Zhang, Liang Ye, Hao Xu, Qin Zhou, Bin Tan, Qin Yi, Liang Yan, Min Xie, Yin Zhang, Jie Tian, Jing Zhu

**Affiliations:** 1 Department of Pediatric Research Institute, Ministry of Education Key Laboratory of Child Development and Disorders, National Clinical Research Center for Child Health and Disorders (Chongqing), China International Science and Technology Cooperation base of Child development and Critical Disorders, Children’s Hospital of Chongqing Medical University, Chongqing, Box 136, No. 3 Zhongshan RD, Yuzhong district, Chongqing, 400014 People’s Republic of China; 2 Chongqing Key Laboratory of Pediatrics, Chongqing, People’s Republic of China; 3 Department of Clinical Laboratory, Ministry of Education Key Laboratory of Child Development and Disorders, National Clinical Research Center for Child Health and Disorders (Chongqing), China International Science and Technology Cooperation base of Child development and Critical Disorders, Children’s Hospital of Chongqing Medical University, Chongqing, People’s Republic of China; 4 Department of Cardiovascular (Internal Medicine), Ministry of Education Key Laboratory of Child Development and Disorders, National Clinical Research Center for Child Health and Disorders (Chongqing), China International Science and Technology Cooperation base of Child development and Critical Disorders, Children’s Hospital of Chongqing Medical University, Chongqing, People’s Republic of China

**Keywords:** Nuclear factor erythroid 2 p45-related factor 2 (NRF2), Human induced pluripotent stem cell-derived cardiomyocytes (hiPSC-CMs), Metabolism

## Abstract

**Background:**

Human induced pluripotent stem cell-derived cardiomyocytes (hiPSC-CMs) hold great promise for regenerative medicine and in drugs screening. Despite displaying key cardiomyocyte phenotypic characteristics, they more closely resemble fetal/neonatal cardiomyocytes and are still immature; these cells mainly rely on glucose as a substrate for metabolic energy, while mature cardiomyocytes mainly employ oxidative phosphorylation of fatty acids. Studies showed that the alteration of metabolism pattern from glycolysis to oxidative phosphorylation improve the maturity of hiPSC-CMs. As a transcription factor, accumulating evidences showed the important role of NRF2 in the regulation of energy metabolism, which directly regulates the expression of mitochondrial respiratory complexes. Therefore, we hypothesized that NRF2 is involved in the maturation of hiPSC-CMs.

**Methods:**

The morphological and functional changes related to mitochondria and cell maturation were analyzed by knock-down and activation of NRF2.

**Results:**

The results showed that the inhibition of NRF2 led to the retardation of cell maturation. The activation of NRF2 leads to a more mature hiPSC-CMs phenotype, as indicated by the increase of cardiac maturation markers, sarcomere length, calcium transient dynamics, the number and fusion events of mitochondria, and mitochondrial respiration. Bioinformatics analysis showed that in addition to metabolism-related genes, NRF2 also activates the expression of myocardial ion channels.

**Conclusions:**

These findings indicated that NRF2 plays an important role in the maturation of hiPSC-CMs. The present work provides greater insights into the molecular regulation of hiPSC-CMs metabolism and theoretical basis in drug screening, disease modeling, and alternative treatment.

## Background

Because of the irreparability of cardiac tissue and the scarcity of organs for donation, human induced pluripotent stem cell-derived cardiomyocytes (hiPSC-CMs) hold great potential for the treatment of myocardial injury [1]. However, multiple studies indicated that although hiPSC-CMs demonstrate obvious spontaneous beating; their metabolism, morphology, and structural functions are similar to those of embryonic immature cardiomyocytes [2, 3]. The differences between mature and immature cardiomyocytes are manifested in many cellular aspects [4]. Compared with the morphology of embryonic cardiomyocytes, mature ones are larger, with significantly increased number of nucleus and more uniform cell pulsation. In the cellular ultrastructure, the number, volume, fusion, and cristae density of mitochondria are increased in mature hiPSC-CMs [5]. The mitochondria of embryonic cardiomyocytes are underdeveloped; therefore, these cells mainly rely on glucose as a substrate for metabolic energy, while mature cardiomyocytes mainly employ oxidative phosphorylation of fatty acids to fulfill the rising demand for energy [6]. Studies showed that the alteration of metabolism pattern from glycolysis to oxidative phosphorylation improves the maturity of hiPSC-CMs [7, 8].

The cap’n’collar basic leucine zipper (CNC-bZip) transcription factor, nuclear factor erythroid 2 p45-related factor 2 (NRF2), is the major regulator of cellular redox homeostasis. NRF2-targeted genes generate a large network of antioxidant enzymes, proteins, and transcriptional factors involved in energy metabolism, mitochondrial function, drug metabolism, proteasome degradation, and DNA repair [9]. In recent years, accumulating evidences showed the important role of NRF2 in the regulation of energy metabolism. A study by Ramachandra et al. showed that the expression of NRF2 significantly elevated in hiPSC-CMs which matured by fatty acids (FA) [10]. In addition, in the mitochondria of neurons and mouse embryonic fibroblasts, loss of NRF2 leads to mitochondrial respiration damage and decreased ATP levels and mitochondrial membrane potential, whereas genetic activation of NRF2 increases the rate of respiration, the efficiency of oxidative phosphorylation, and ATP levels [11]. It has been determined in different experimental systems that NRF2 directly regulates the expression of mitochondrial respiratory complexes such as ATP synthase subunit α, NDUFA4, and cytochrome C oxidase subunits COX2 and COX4B [12, 13]. In addition to mitochondrial respiration, NRF2 also promotes fatty acid β-oxidation metabolism [14]. Moreover, some studies show that NRF2 protects myocardium from damage due to its effects on anti-oxidative stress and promotion of mitochondrial aerobic respiration [15]. Therefore, we hypothesized that NRF2 is involved in the maturation of hiPSC-CMs. In this study, the morphological and functional changes related to mitochondria and cell maturation were analyzed by knock-down and activation of NRF2. The results showed that the inhibition of NRF2 led to the retardation of cell maturation, while the activation of NRF2 significantly promoted the cell maturation, which indicated the essential function of NRF2 in the maturation of hiPSC-CMs.

## Materials and methods

### Culture and cardiac differentiation of human induced pluripotent stem cell

Undifferentiated human epithelial cells of the urine-derived iPS cells (hiPSCs), derived from CELLAPY Biological Technology (China), were maintained on Matrigel (BD Biosciences)-coated 6-well cell culture plates with PGM1 (CELLAPY Biological Technology). For cardiac cell differentiation, hiPSCs at ~ 80% confluence was dissociated using EDTA (CELLAPY Biological Technology) and plated onto the Matrigel-coated 6-well plates (Corning) at a density of 4 × 10^5^ cells/well. When the cells reached 90–98% confluency, typically 4 days after passage, the medium was changed to RPMI plus B27 without insulin (Life Technologies) supplemented with 6 μM GSK3-β inhibitor CHIR99021 (CHIR) (Selleck). Forty-eight hours later, the medium was completely replaced with RPMI/B27 medium without insulin and incubated for 24 h. Then, the RPMI/B27 medium without insulin was exchanged with 5 μM Wnt inhibitor IWP2 (Selleck). On day 5, the medium was refreshed with RPMI/B27 medium without insulin and incubated until day 7. The medium was then exchanged every 3 days with RPMI/B27 medium (Life Technologies). During day 13 to 16, metabolic purification of the cardiomyocytes was performed by replacing the medium with RPMI medium without glucose and supplemented with B27 and 4 mM sodium L-lactate (Sigma) [16]. On day 18 of cardiac cell differentiation, the cells were dissociated with TrypLE Express Enzyme (Gibco) and seeded into Matrigel-coated 12-well plates (Corning) in RPMI/B27 medium. Then, cell cultures containing > 90% cardiac troponin T-positive CMs (confirmed by flow cytometry) were obtained.

### siRNA transfection

The siRNAs used in this study were stealth-siRNA human NFE2L2 (NRF2, Thermo Fisher Scientific, MA), human KEAP1 siRNA (RIB BIO, MO), and negative control siRNA. The siRNAs were transfected into CMs with Lipofectamine RNAiMAX Reagent (Thermo Fisher Scientific, MA) at a concentration of 70 nM in each well of a 12-well plate according to the manufacturer’s recommendations. siRNA transfection was performed on day 24 of cardiac cell differentiation, and the cells were analyzed 3 days later.

### Immunostaining and confocal microscopic imaging

#### Immunofluorescence staining

Following 3 washes with PBS, cells (plated on a 1 × 1 cm glass slide) were fixed for 20 min with 4% paraformaldehyde and washed again with PBS. The samples were then incubated for 10 min at room temperature with 0.5% Triton X-100 in PBS and blocked with 5% bovine serum albumin (BSA) in PBS for 30 min at room temperature. Primary antibodies were diluted in 5% BSA in PBS and incubated overnight at 4 °C. Following 3 washes with 0.5% Triton X-100 in PBS, the samples were incubated with secondary antibodies diluted in 5% BSA for 60 min at 37 °C. The samples were then washed, incubated with Hoechst 33342 (Beyotime, China) in PBS for 30 min at 37 °C and washed again before imaging. Immunofluorescence staining was imaged using an A1R confocal microscope (Nikon, Japan). The following antibodies were used in this study: anti-SOX2, anti-Nanog (Proteintech, China), anti-cTnT, anti-CX43 (Abcam, China), and anti-α-actinin (Proteintech, China). The secondary antibodies were goat anti-rabbit Cy3, rabbit anti-mouse Cy3 (CWBIO, Beijing, China), and goat anti-rabbit 488 (ZSGB-Bio, Beijing, China). Sarcomere lengths and nuclear numbers were measured by ImageJ software. *n* > 10 cells per condition, three biological replicates. The lengths of ten sarcomeres from each cell were measured and averaged for each condition.

#### Mitochondrial staining

hiPSC-CMs were stained with either JC-1 dye (Beyotime, China) or prewarmed MitoTracker Red (0.2 μM; Beyotime, China) for 25 min at 37 °C according to the manufacturers’ instructions. For mitochondrial staining, the nucleus was stained first. Ten microliters Hoechst dye was added to 1 ml of medium, incubated for 16 min at 37 °C, and washed three times with PBS. Then, the mitochondria were stained. The staining and fluorescence intensity of the cells was evaluated using an A1R confocal microscope (Nikon, Japan). The exact amount of ATP is determined by an ATP determination kit (Beyotime, China) according to the manufacturer’s instructions.

#### Calcium imaging

Following 3 washes with D-PBS, 0.3 mM Fluo-4 AM (Beyotime) was added to the cells for 40 min at 37 °C, and then, the cells were washed with D-PBS for 3 times. The cells were incubated at 37 °C for 20 min before observation with A1R confocal microscope (Nikon, Japan). Videos were captured in continuous shooting mode using 488 channels.

#### Ethynyl-20-Deoxyuridine (EdU) cell proliferation assay

Cell proliferation was assessed with a BeyoClick™ EdU cell proliferation kit with Alexa Fluor 488 (Beyotime, China). After 6.67 μM EdU was added to cells, the cells were cultured for 15 h at 37 °C to incorporate the reagent. After the cTnT of hiPSC-CMs were labeled using immunofluorescence, the cells then were incubated in Click Additive Solution and protected from light, washed three times with 3% BSA in PBS, and stained with Hoechst 33342. Fluorescence images were obtained using an A1R confocal microscope (Nikon, Japan). The ratio of proliferating cells was determined by ImageJ software.

### Flow cytometry

Following 3 washes with D-PBS, cells were fixed for 20 min with 4% (vol/vol) paraformaldehyde. The samples were then incubated for 10 min at room temperature with 0.5% Triton X-100 in PBS, blocked for 30 min at room temperature with 5% bovine serum albumin in PBS, and labeled with rabbit anti-cTnT/AF488 (Bioss antibodies) in PBS. The cells were analyzed with a BD FACSCanto analyzer (BD Biosciences). Data analysis was performed using FlowJo software.

### Analysis of mtDNA copy number

To evaluate mtDNA copy number, genomic and mitochondrial DNA was extracted with genomic DNA extraction kit (BioFlux). The extracted DNA was used as template for quantitative PCR (QuantStudio 3; Thermo Fischer Scientific) with a TB Green Premix Ex Taq kit (Roche, Basel, Switzerland) according to the manufacturer’s instructions. The mitochondrial DNA (mt-ND1) to nuclear DNA (β-globin) ratio was calculated as the mtDNA copy number. Error bars indicate the standard deviation of triplicate measurements of three biological samples.

### Transmission electron microscopy (TEM)

Cells with a density greater than 1 × 10^6^ were collected in a 1.5-ml centrifuge tube, fixed with 2.5% glutaraldehyde for 24 h, and observed under a transmission electron microscope (TEM, Hitachi-7500, Japan) after rinsing, dehydration, soaking, and embedding.

### Mitochondrial function assay

#### Mitochondrial respiration

The mitochondrial oxygen consumption rate (OCR) was assessed by an Agilent Seahorse XF Cell Mito Stress Test Kit and a 24-well XF cell culture microplate (Agilent Technologies, CA, USA) with nearly 1 × 10^5^ cells added to each well. Before the assay, the cells were maintained on Seahorse XF base medium containing 1 M glucose, 100 mM pyruvate, and 200 mM L-glutamine. During OCR assessment, oligomycin (1.5 μM), FCCP (2 μM), and antimycin A/rotenone (0.5 μM) were added to the system. Basal respiration, proton leakage, maximal respiration, ATP product, and spare respiratory capacity were measured in a XF24 analyzer.

#### FAO assay

Fatty acid oxidation (FAO) was assessed by an Agilent Seahorse XF Substrate Oxidation Stress Test Kit, same with mitochondrial stress test. Except for OCR assessment, there are four reagents Etomoxir (Eto, 4 μM), oligomycin (1.5 μM), FCCP (2 μM), and antimycin A/rotenone (0.5 μM) were added to the system, other operation of the experiment is similar to Mito Stress Test. Basal respiration, proton leakage, maximal respiration, ATP product, and spare respiratory capacity were measured in a XF24 analyzer.

#### Glycolysis analysis

The glucose and lactic acid content detection kits (Solarbio, China) were used to detected glucose content and lactic acid content of cells, respectively. According to the original glucose and lactic acid content in the medium, the glucose consumption and lactic acid production are calculated, and the results are calibrated with protein content.

### Western blot analysis

Western blot experiments were performed to determine the protein expression in cell lysates. The protein concentration was quantified with BCA protein quantitation assay (KeyGen Biotech, China). Equal protein amounts (30 μg) were loaded onto SDS-PAGE gels and transferred to PVDF membranes (Millipore Sigma, China). Phosphorylated protein or common protein was blocked for 2 h at room temperature in 5% BSA or 5% skim milk in TBS with 0.05% Tween-20. Protein bands were probed overnight with the suitable primary antibody at 4 °C. Proteins were visualized using HRP-conjugated secondary antibody and a chemiluminescent detection kit (Millipore, USA). The amount of target protein was calculated by gray scanning.

### RNA extraction and quantitative PCR

Total RNA was extracted with RNAiso Reagent (TaKaRa, Japan), and the RNA solution was obtained after chloroform extraction, isopropanol precipitation, ethanol washing, and RNase-free water dissolution. cDNA synthesis was completed with PrimeScript™ RT reagent kit with gDNA Eraser (TaKaRa). mRNA expression was measured by RT-PCR using a TB Green Premix Ex Taq kit (Roche, Basel, Switzerland) on a QuantStudio 3 Real-Time PCR System (Thermo Fischer Scientific). The reaction conditions were as follows: initial denaturation at 95 °C for 10 min; 39 cycles of denaturation at 95 °C for 5 s, annealing, and extension at 60 °C for 30 s; and a melt curve reaction cycle. The fold change in expression was calculated with 2^−ΔΔCT^ method with GAPDH RNA as the endogenous control.

### Statistical analysis

All experiments showed in this research have been repeated more than three times. The data were analyzed by unpaired *t*-tests or Mann-Whitney *U* test after a demonstration of homogeneity of variance with the *F* test. Differences at a *P* value of < 0.05 were considered significant. Statistical analysis was performed by GraphPad Prism, version 7.

## Results

### Characteristics of the hiPSCs and hiPSC-CMs

**Fig. 1:**
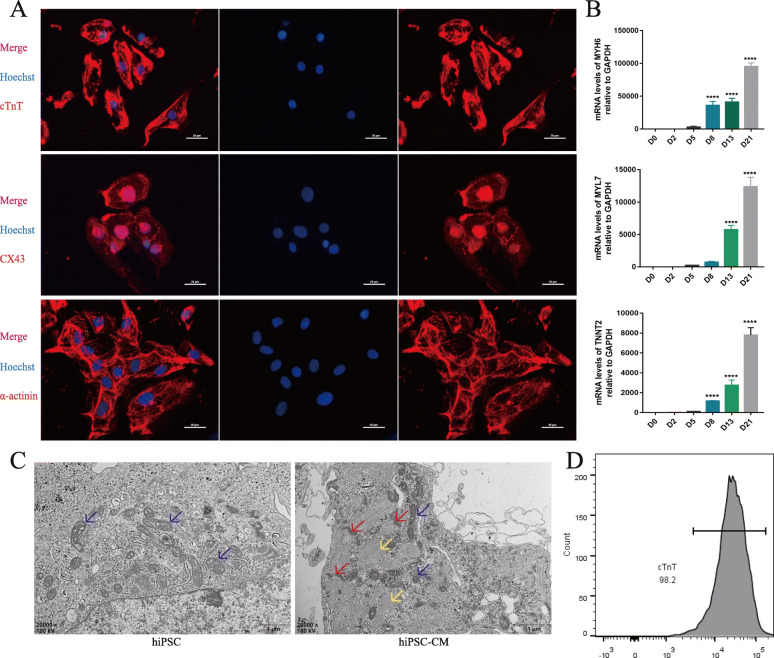
Characterization of hiPSC-CMs. **a** Immunostaining of hiPSC-CMs for cTnT, CX43, and α-actinin. **b** Q-PCR of cardiac marker genes MYH6, MYL7, and TNNT2 during differentiation. The abscissa represents the number of days of differentiation. *n* = 3; the means ± SEM are shown. ****P < 0.0001. **c** TEM images of hiPSC and hiPSC-CMs, myofibrils (yellow arrow), Z-disks (red arrow), and mitochondria (blue arrow). **d** Flow cytometry analysis of the hiPSC-CMs by cTnT measurements demonstrated cardiac cell purity greater than 98%


The process of hiPSCs differentiation into cardiomyocytes (hiPSC-CMs) is shown in Fig. S1A. By regulating Wnt signal at different time points during differentiation, cardiomyocytes capable of spontaneous beating were obtained on the 13th day of induction (Movie S1) [17, 18]. On day 20 of cardiac cell differentiation, the positive rate of cTnT in the hiPSC-CMs was more than 98%, as determined by flow cytometry (Fig. 1d). Pluripotency of hiPSC was further confirmed by immunocytochemistry (ICC) assay (Fig. S1B) and Q-PCR measurements (Fig. S1C, D). As shown in Supplement Fig. 1C and D, during the differentiation process, the mRNA expression levels of pluripotent markers were significantly decreased. In addition, the expression of cardiac marker in differentiated hiPSC-CMs was observed with immunofluorescence (Fig. 1a), and the expression levels was gradually increased with differentiation (Fig. 1b). Transmission electron microscopy showed that the differentiated hiPSC-CMs displayed a myocardial-specific sarcomere structure (Fig. 1c). These results demonstrated that the successfully differentiated cardiomyocytes were obtained.

**Additional file 2: Movie S1**: Spontaneously beating of hiPSC-CMs.

### Inhibition of NRF2 suppresses hiPSC-CMs maturation

**Fig. 2:**
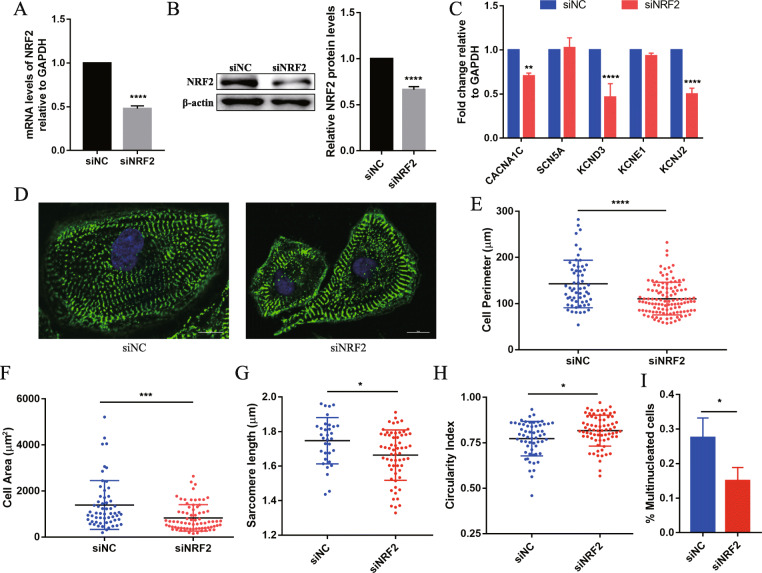
NRF2 is required for hiPSC-CMs morphology and structure maturation. **a** Q-PCR and **b** Western blotting (left) and quantification (right) were used to detect the expression of NRF2 in the siNRF2 and siNC hiPSC-CMs. *n* = 3. **c** mRNA expression of ion channels in siNRF2 and siNC hiPSC-CMs. The gene expression is shown normalized first to GAPDH and then to siNC. **d** α-Actinin (green) and Hoechst (blue) staining of representative siNRF2 and siNC hiPSC-CMs. Scale bar = 10 μm. **e**–**i** Compared with the siNC hiPSC-CMs, the siNRF2 hiPSC-CMs showed a significant decrease in **e** cell perimeter, **f** cell area, **g** sarcomere length, and **i** multinucleated cell ratio (*n* = 3) and **h** an increase in the circularity index (*n* > 10 cells per condition, three biological replicates). The means ± SEM are shown. *P < 0.05, **P < 0. 01, ***P < 0. 001, ****P < 0. 0001

To test the role of NRF2 in hiPSC-CMs, the *NRF2* gene was knocked down by siRNA. NRF2 expression was significantly down-regulated in the NRF2-inhibited hiPSC-CMs (siNRF2) compared with that in the control hiPSC-CMs (siNC) by Q-PCR and Western blotting (Fig. 2a, b). Firstly, the effects of NRF2 knock-down on the morphology and function of hiPSC-CMs were investigated. Encouragingly, the inhibition of NRF2 caused a significant decrease of cardiac maturation markers, including potassium (KCND3 and KCNJ2) and calcium (CACNA1C, an L-type Ca channel protein, correlated with the inward depolarizing current) ion channel protein-coding genes (Fig. 2c). The results further inspired us to characterize multiple parameters modulated during the process of hiPSC-CMs maturation. Therefore, α-actinin (Z-disk protein) was stained to visualize the siNC and siNRF2 hiPSC-CMs (*n* > 15 cells per condition, three biological replicates) (Fig. 2d). Significant decreases in cell perimeter (siNRF2, 110.7 ± 3.242 μm vs. siNC, 142.5 ± 6.676 μm; *P* < 0.0001), cell area (siNRF2, 834.5 ± 66.34 μm^2^ vs. siNC, 1391 ± 138 μm^2^; *P* < 0.001), and sarcomere length (siNRF2, 1.663 ± 0.018 μm vs. siNC, 1.747 ± 0.023 μm; *P* < 0.01) were observed in the siNRF2 hiPSC-CMs compared with the siNC hiPSC-CMs (Fig. 2e–g). The circularity index [4π area/(perimeter)^2^] (“0” represents a theoretical minimum for perfect rod-shaped cells, with “1” for perfectly round cells) increased from 0.77 ± 0.013 in siNC hiPSC-CMs to 0.82 ± 0.01 in the siNRF2 hiPSC-CMs, which indicated that the cells transformed into a round immature form (Fig. 2h). In addition, although human CMs do not proliferate, they are capable of DNA synthesis without nuclear division or nuclear division without cytokinesis, thereby increasing in ploidy (8 N) and size (hypertrophy) [19, 20]. Hoechst staining was employed to analyze the number of nuclei, and the results showed that compared with the siNC cells, the proportion of multinuclear cells in siNRF2 hiPSC-CMs was significantly reduced (siNRF2, 0.1504 ± 0.022 vs. siNC, 0.2754 ± 0.033; *P* < 0.05).

In addition, the calcium transient kinetics were assessed during contraction of hiPSC-CMs with Fluo-4 AM. These results showed that reduced peak amplitude, upstroke, and decay velocities were obtained in the siNRF2 hiPSC-CMs compared with the siNC hiPSC-CMs (Fig. S2A-D), indicating that the calcium handling system was weakened. These results demonstrated that the morphologic and functional maturation of hiPSC-CMs was arrested after NRF2 inhibition.

### Inhibition of NRF2 suppresses hiPSC-CM mitochondrial maturation

To further analyze the effect of NRF2 on hiPSC-CM mitochondrial maturation, the morphology, number, and function of mitochondrial were evaluated after NRF2 inhibition. Research has shown that the abundance of mitochondria is increased with the maturity of the myocardium to meet higher energy demands [21]. Thus, we measured the abundance of mitochondrial DNA (mtDNA) by normalizing mtDNA to genomic nuclear DNA (gDNA). The mtDNA/gDNA ratio was significantly lower in the siNRF2 hiPSC-CMs than that in the siNC cells (siNRF2, 1.39 ± 0.012 vs. siNC, 1.45 ± 0.013; *P* < 0.05) (Fig. S3A). Accumulating evidence suggests that mitochondrial fusion plays a central role in the development of cardiomyocytes [22]. Therefore, we examined mitochondrial of live cells stained with MitoTracker Red by confocal laser scanning microscopy. As shown in Fig. S3B, the shape of mitochondria was transformed from an elongated to a punctiform phenotype, illustrating the reduction in mitochondrial fusion in the NRF2-inhibited cells compared with the siNC cells. The JC-1 fluorescent probe is used as an indicator of mitochondrial membrane potential (ΔΨm), which is also an important parameter used to assess the functional state of these organelles. The inhibition of NRF2 resulted in a reduction in mitochondrial membrane potential compared to that in the siNC cells (siNRF2, 0.557 ± 0.029 vs. siNC, 0.849 ± 0.056; *P* < 0.0001) (Fig. S3C, D).

**Fig. 3:**
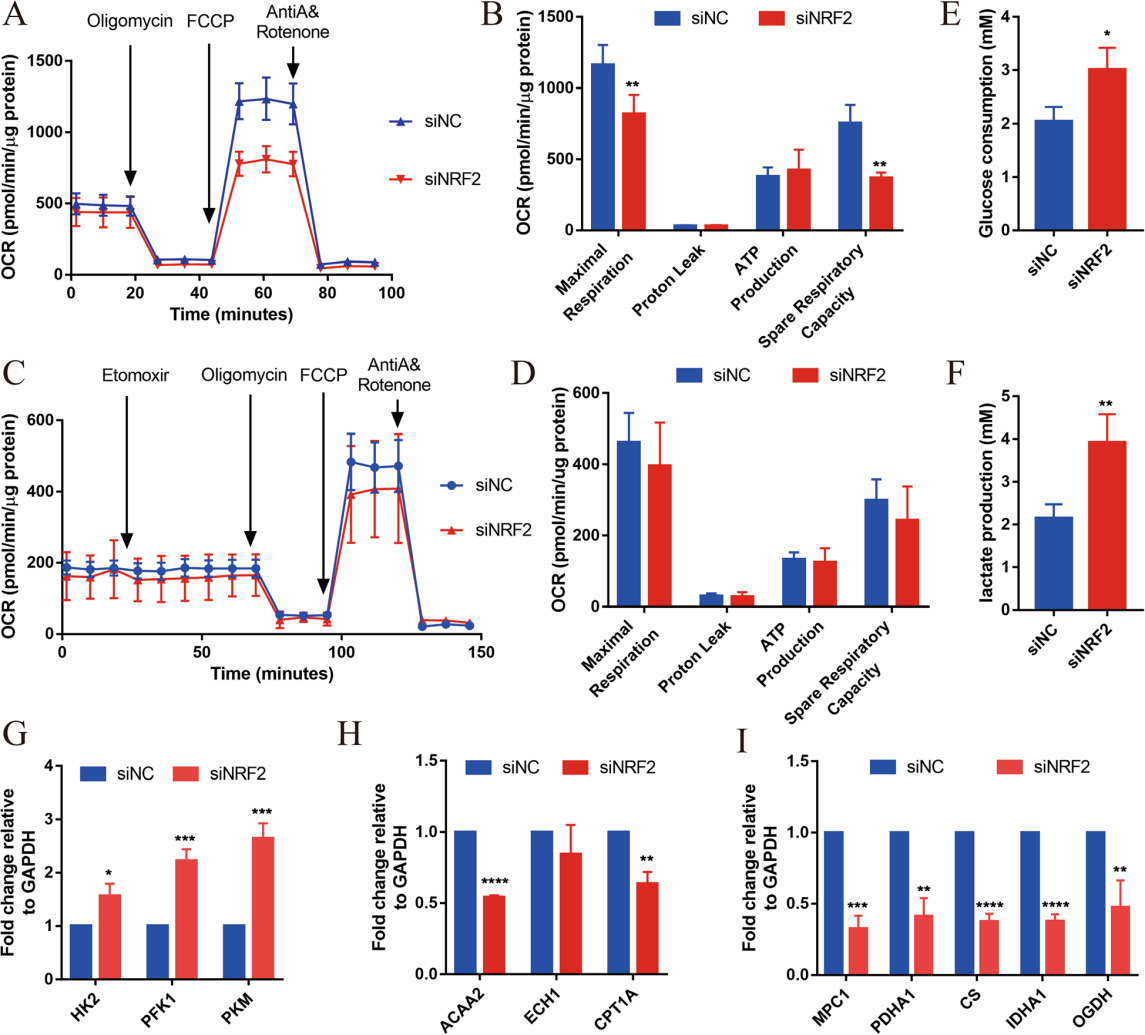
NRF2 is required for hiPSC-CM mitochondrial metabolism maturation. **a** Representative mitochondrial respiration in siNRF2 and siNC hiPSC-CMs after incubation with the ATP synthase inhibitor oligomycin, the respiratory uncoupler carbonyl cyanide-p-trifluoromethoxyphenylhydrazone (FCCP), and the respiratory chain blockers rotenone and antimycin A. **b** Quantification of maximal respiration capacity, proton leakage, ATP production, and spare respiratory capacity in siNRF2 and siNC hiPSC-CMs (*n* = 4 for each group). **c** Representative fatty acid oxidation in siNRF2 and siNC hiPSC-CMs after incubation with the specific inhibitor of carnitine palmitoyl transferase 1A (CPT1A) Etomoxir, oligomycin, FCCP, and rotenone and antimycin A. **d** Quantification of maximal respiration capacity, proton leakage, ATP production, and spare respiratory capacity in siNRF2 and siNC hiPSC-CMs (*n* = 4 for each group). The means ± SEM are shown. **p < 0.01. Glucose consumption (**e**) and lactic acid generation (**f**) of siNRF2 and siNC hiPSC-CMs were detected by kit. *n* = 3. Relative expression levels of the key metabolic genes in glycolysis (**g**), fatty acid β-oxidation (**h**), and aerobic oxidation (**i**). *n* = 3. The means ± SEM are shown. *P < 0.05, **P < 0.01, ***P < 0.001, ****P < 0.0001

We also evaluated mitochondrial function in NRF2-inhibited hiPSC-CMs by the XFe24 Cell Mito Stress Test (Seahorse Bioscience) and Substrate Oxidation Stress Test Kit (Fig. 3a, c). The results of oxygen consumption rate (OCR) were normalized based on the protein content. In the Mito Stress Test, the basal OCR was significantly lower in the siNRF2 hiPSC-CMs than in siNC hiPSC-CMs (Fig. 3a). The maximum respiration rate and spare respiratory capacity were also significantly lower in the siNRF2 hiPSC-CMs than in the siNC hiPSC-CMs (Fig. 3b). However, no significant difference in ATP production was observed between the siNRF2 hiPSC-CMs and siNC hiPSC-CMs (Fig. 3b). Similarly, the level of proton leakage, a sign of mitochondrial damage and a mechanism to regulate mitochondrial ATP production, did not significantly differ between the siNRF2 hiPSC-CMs and siNC hiPSC-CMs (Fig. 3b). In the Substrate Oxidation Stress Test, Etomoxir, a specific inhibitor of carnitine palmitoyl transferase 1A (CPT1A), was used to specifically inhibit mitochondrial FAO. After Etomoxir treatment, the maximum respiration rate, spare respiratory capacity, ATP production, and proton leakage did not decrease in NRF2-inhibited hiPSC-CMs comparing with siNC hiPSC-CMs (Fig. 3d), indicating the fatty acid oxidation energy of siNRF2 hiPSC-CMs did not elevate. While the glucose consumption and lactic acid generation of siNRF2 hiPSC-CMs significantly increased (Fig. 3e, f). This result suggests that the knock-down of NRF2 led to the decrease of mitochondria respiration and increase of glycolysis.

In addition, we also tested the expression of genes related to glycolysis, oxidative phosphorylation, and fatty acid β-oxidation. Hexokinase (HK2), phosphofructokinase (PFK), and pyruvate kinase (PKM) used as surrogates for glycolysis rates were significantly increased in siNRF2 hiPSC-CMs compared with the control cells (Fig. 3g). In contrast with the high expression of these glycolysis genes, the expression of acetyl-CoA acyltransferase 2 (ACAA2) and carnitine palmitoyl transferase 1A (CPT1A), which encode key mitochondrial proteins involved in the β-oxidation of fatty acids, were dramatically reduced (Fig. 3h). In addition, the expression levels of mitochondrial pyruvate carrier 1 (MPC1), pyruvate dehydrogenase E1 subunit alpha 1 (PDHA1), citrate synthase (CS), isocitrate dehydrogenase (IDHA1), and oxoglutarate dehydrogenase (OGDH), involved in aerobic oxidation regulation, were significantly reduced in siNRF2 hiPSC-CMs compared with the control cells (Fig. 3i). These data indicate that NRF2 is essential for the mitochondrial maturation of hiPSC-CMs.

### Activation of NRF2 enhances hiPSC-CMs maturation

**Fig. 4:**
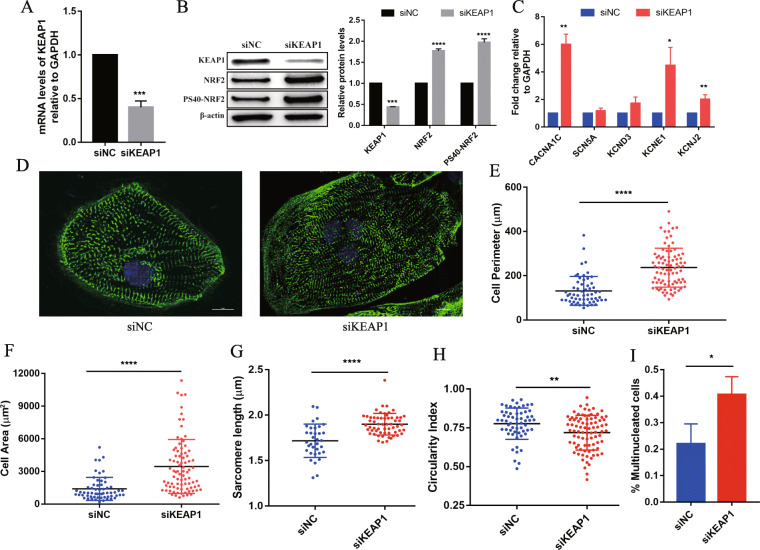
NRF2 promotes the morphology and structural maturation of hiPSC-CMs. **a** Q-PCR to detect the expression of KEAP1 in siKEAP1 and siNC hiPSC-CMs. **b** Western blot (left) and quantification (right) to detect the expression of KEAP1, NRF2, and phosphorylated NRF2 (PS40-NRF2) in siKEAP1 and siNC hiPSC-CMs. *n* = 3. **c** mRNA expression of ion channels in siKEAP1 and siNC hiPSC-CMs. Gene expression is normalized first to GAPDH and then normalized to siNC. **d** α-Actinin (green) and Hoechst (blue) staining of representative siKEAP1 and siNC hiPSC-CMs (scale bar = 10 μm). **e**–**i** Compared with siNC cells, siKEAP1 cells showed a significant increase in **e** perimeter, **f** area, **g** sarcomere length, and **i** multinucleation ratio (*n* = 3) and **h** a decrease in the circularity index (*n* > 10 cells per condition, three biological replicates). The means ± SEM are shown. *P < 0.05, **P < 0.01, ***P < 0.001, ****P < 0.0001

Under normal physiological conditions, NRF2 forms an E3 ubiquitin ligase complex consisting of cullin-3 (Cul3) with Kelch-like ECH-related protein 1 (KEAP1) outside the nucleus and is ubiquitinated and degraded. When dissociates from Keap1 and is translocated to the nucleus, NRF2 forms a heterodimer with the small Maf protein, binds to the antioxidant response element (ARE) in the promoter region of the target gene, and initiates transcription [23–25]. To further examine the effect of NRF2 on hiPSC-CMs maturation, we activated NRF2 by knocking down *KEAP1* expression. After transfection of siKEAP1, the mRNA and protein expression levels of KEAP1 in hiPSC-CMs were significantly reduced (Fig. 4a, b), while the total protein expression levels of NRF2 and phosphorylated NRF2 (PS40-NRF2) were significantly increased (Fig. 4b), indicating that KEAP1 knock-down was effective and NRF2 was successfully activated. Then, the expression levels of the ion channel-related genes that activated by NRF2 in hiPSC-CMs were tested. These results showed that after NRF2 activation, the expression of genes encoding sodium (SCN5A), potassium (KCND3, KCNJ2 and KCNE1), and calcium channels (CACNA1C) was significantly increased, especially for CACNA1C, which was 6-fold higher than that of siNC (Fig. 4c). In addition, immunocytochemical analyses with α-actinin for Z-disk protein staining and Hoechst for nuclear counterstaining showed that NRF2 activation led to maturation changes in hiPSC-CMs. We observed a significant increase in cell perimeter (siKEAP1, 236.6 ± 9.46 μm vs. siNC, 131.6 ± 8.48 μm, *P* < 0.0001), cell area (siKEAP1, 3452 ± 269 μm^2^ vs. siNC, 1392 ± 138 μm^2^, *P* < 0.0001), sarcomere length (siKEAP1, 1.9 ± 0.015 μm vs. siNC, 1.718 ± 0.032 μm, *P* < 0.0001), and proportion of cells with multiple nuclei (siKEAP1, 0.4064 ± 0.039 vs. siNC, 0.2202 ± 0.044, *P* < 0.05) in NRF2-activated cells compared with siNC (Fig. 4d–h). Moreover, NRF2 activation resulted in a decreased circularity index (siKEAP1, 0.719 ± 0.012 vs. siNC, 0.777 ± 0.013 *P* = 0.0017) (Fig. 4i). These data indicated that NRF2 promoted the morphology maturation of hiPSC-CMs.

**Fig. 5:**
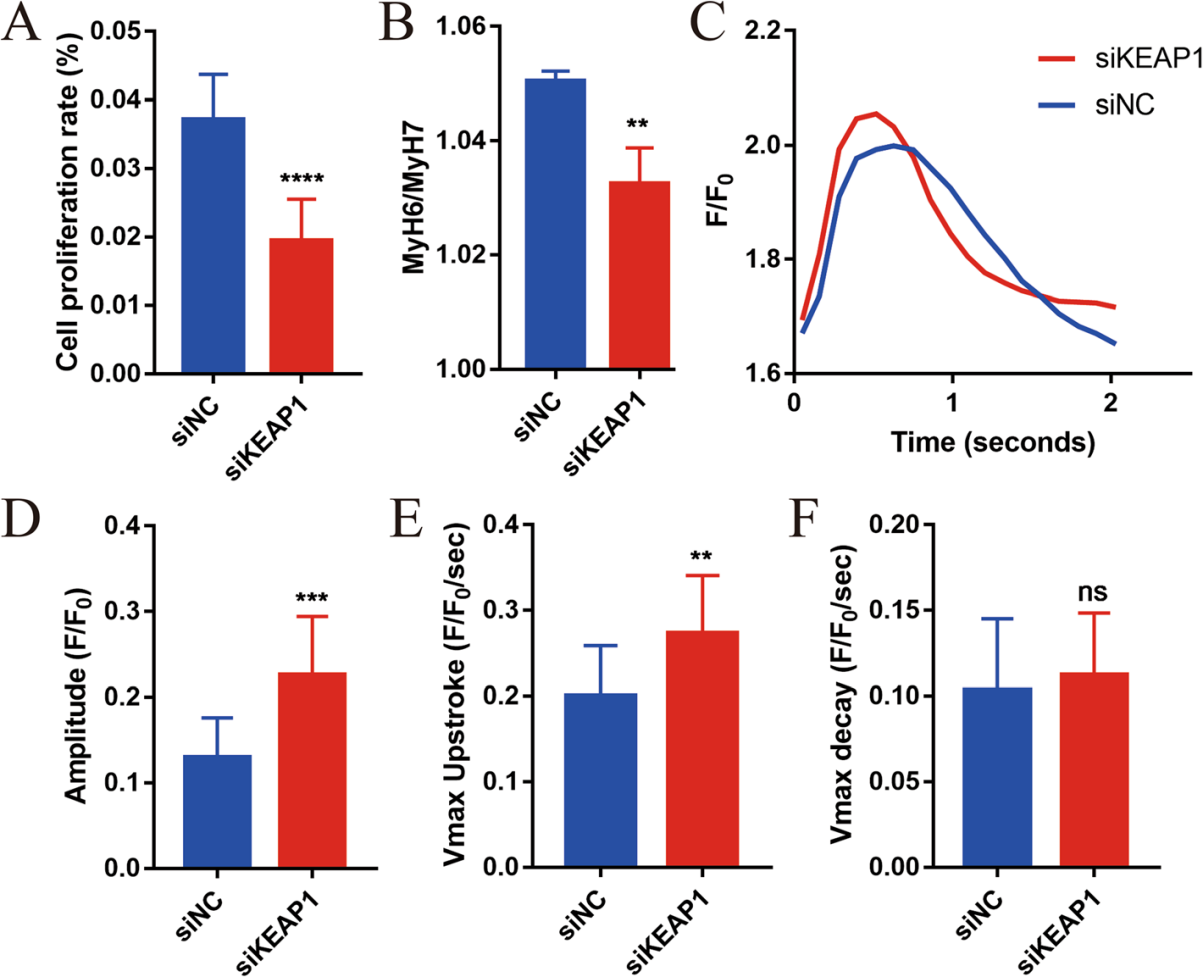
NRF2 promotes function maturation of hiPSC-CMs. **a** Quantification of the percentage of siKEAP1 and siNC hiPSC-CMs proliferating, as measured by EdU (each biological replicate *n* > 5, three biological replicates). **b** The proportion of MyH6 to MyH7 was decreased in the siKEAP1 hiPSC-CMs. *n* = 3. **c**, **d** HiPSC-CMs were analyzed by calcium transient kinetics with Fluo-4 AM (*n* > 3 cells per condition, three biological replicates): **c** representative calcium transient and **d** calcium transient amplitude (F/F0). **e** Maximum calcium transient upstroke velocities. **f** Maximum calcium transient decay velocities. The means ± SEM are shown. **P < 0.01, ***P < 0.001, ****P < 0.0001

Cardiomyocytes in the embryonic period undergo a certain level of proliferation, and the proliferative ability is significantly weakened after maturation. EdU was employed to detect the cell proliferation level, and the results showed that the proliferation level of NRF2-activated cells was significantly lower than that of siNC cells (Fig. 5a and Fig. S4A). Studies have reported that myosin heavy chain 6 (MyH6, fast-twitch) converts to myosin heavy chain 7 (MyH7; slow-twitch) during the process of myocardial maturation [26, 27]. We observed a significant decrease in MyH6/MyH7 in the siKEAP1 hiPSC-CMs (siKEAP1, 1.032 ± 0.004 vs. siNC, 1.05 ± 0.001, *P* < 0.01) in NRF2-activated cells compared with control cells (Fig. 5b). Compared with siNC cells, the calcium transient kinetics of NRF2-activated hiPSC-CMs presented significant increases in peak calcium amplitude and faster Ca^2+^ transient upstroke velocities indicating calcium transient kinetics (Fig. 5c–e), which were consisted with elevated CACNA1C levels. These data are indicative of a more mature calcium transport system. However, the decay velocities were not significantly different (Fig. 5f). In summary, the function of hiPSC-CMs became more mature after activation of NRF2.

### Activation of NRF2 enhances hiPSC-CM mitochondrial maturation

Studies have shown that NRF2 promotes mitochondrial biogenesis. Test results showed that the mtDNA copy number was significantly higher in the siKEAP1 hiPSC-CMs than that in the siNC hiPSC-CMs (siKEAP1, 1.445 ± 0.009 vs. siNC, 1.415 ± 0.004, *P* < 0.05) (Fig. S5A). The MitoTracker Red stain showed that mitochondria changed from an elongated to a network phenotype, indicating that the degree of mitochondrial fusion was significantly increased in the NRF2-activated cells compared to that in the control cells (Fig. S5B). The JC-1 test results showed that NRF2 activation resulted in an increase in mitochondrial membrane potential compared to that in siNC cells (siKEAP1, 1.234 ± 0.062 vs. siNC, 0.949 ± 0.037, *P* < 0.001) (Fig. S5C, E). These data suggest that mitochondrial morphology and structure were more mature in NRF2-activated hiPSC-CMs.

**Fig. 6:**
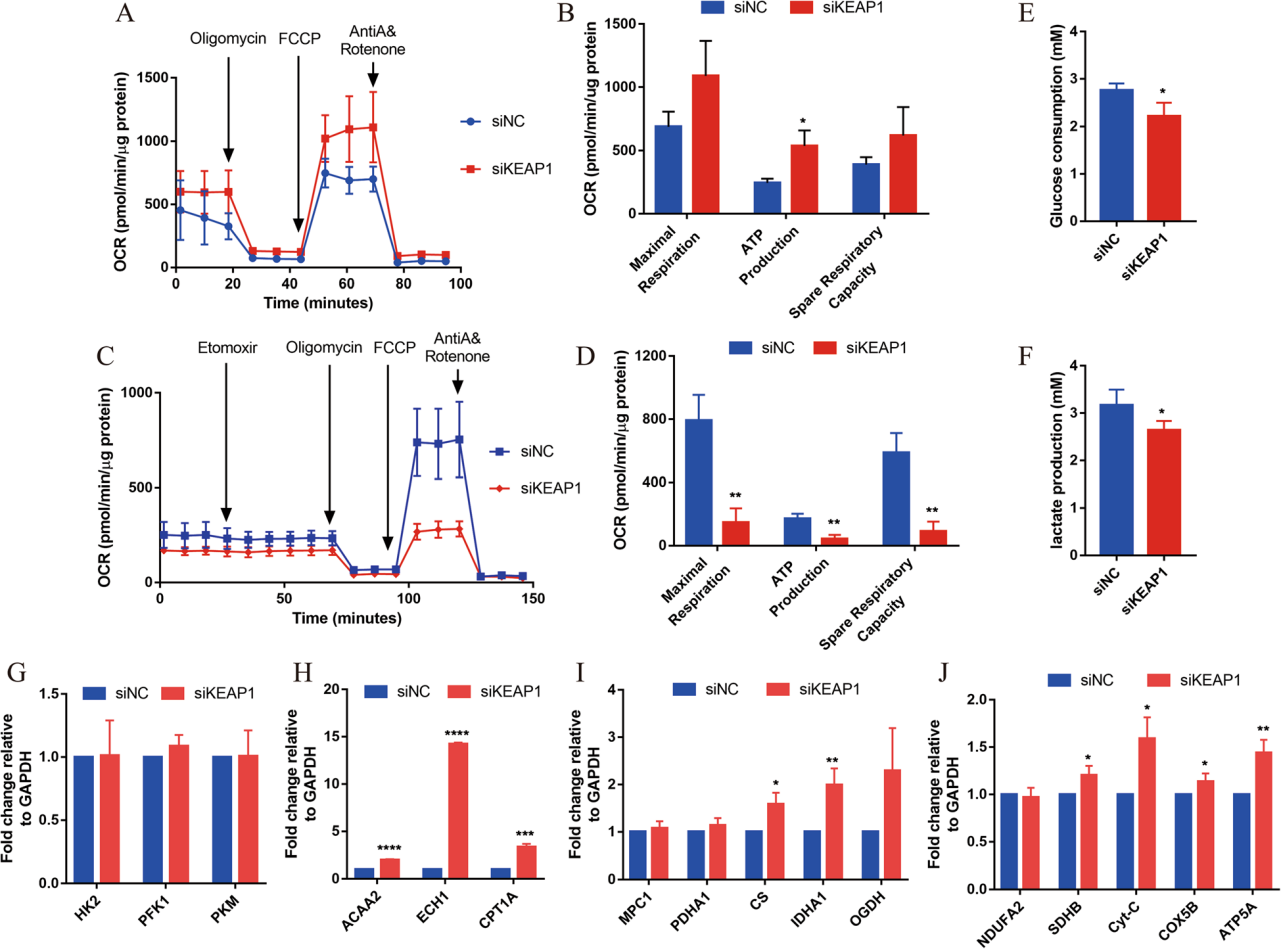
NRF2 promotes hiPSC-CM mitochondrial metabolism maturation. **a** Representative mitochondrial respiration in siKEAP1 and siNC hiPSC-CMs after incubation with oligomycin, FCCP, rotenone, and antimycin A. **b** Quantification of the maximal respiration capacity, ATP production, and spare respiratory capacity in the siKEAP1 and siNC hiPSC-CMs (*n* = 4 for each group). The means ± SEM are shown. *P < 0.05. **c** Representative fatty acid oxidation in siKEAP1 and siNC hiPSC-CMs after incubation with the Etomoxir, oligomycin, FCCP, and rotenone and antimycin A. **d** Quantification of maximal respiration capacity, proton leakage, ATP production, and spare respiratory capacity in siKEAP1 and siNC hiPSC-CMs (*n* = 4 for each group). The means ± SEM are shown. **p < 0.01. Glucose consumption (**e**) and lactic acid generation (**f**) of siNRF2 and siNC hiPSC-CMs were detected by kit. *n* = 3. Relative expression levels of the key metabolic genes in glycolysis (**g**), fatty acid β-oxidation (**h**), aerobic oxidation (**i**), and electron transport chain complex (**j**); *n* = 3; the means ± SEM are shown. *P < 0.05, **P < 0.01, ***P < 0.001, ****P < 0.0001

To further verify whether the function of mitochondria is enhanced after NRF2 activation, the Agilent Seahorse XF Cell Mito Stress Test Kit and Substrate Oxidation Stress Test Kit were employed to test the mitochondrial function of hiPSC-CMs after NRF2 activation. In the Mito Stress Test, the basal OCR was significantly increased in the siKEAP1 hiPSC-CMs than which in the siNC hiPSC-CMs (Fig. 6a), and the production of ATP was also significantly higher in the siKEAP1 hiPSC-CMs, which was consisted with the results determined by an ATP detection kit (Fig. 6b, S5D). In addition, as shown in Fig. 6b, the maximum respiration rate (siKEAP1, 1089 ± 160.5 pmol/min vs. siNC, 689.2 ± 69.45 pmol/min, *P* = 0.0842) and spare respiratory capacity (siKEAP1, 620.4 ± 129.8 pmol/min vs. siNC, 389.1 ± 33.77 pmol/min, *P* = 0.1597) were higher in the siKEAP1 than that in the siNC hiPSC-CMs, but the differences were not statistically significant. And in the Substrate Oxidation Stress Test, the increased fatty acid oxidation energy of siKEAP1 hiPSC-CMs was shown by the decrease in oxygen consumption rate upon incubation with etomoxir (Fig. 6c). The maximum respiration rate, spare respiratory capacity, and the production of ATP were lower in the siKEAP1 than that in the siNC hiPSC-CMs (Fig. 6d). In addition, the glucose consumption and lactic acid generation was significantly decreased in the siKEAP1 hiPSC-CMs than which in the siNC hiPSC-CMs (Fig. 6e, f). Taken together, NRF2-activated hiPSC-CMs exhibited increasing mitochondrial respiratory and inhibited glycolysis compared with the siNC hiPSC-CMs.

It has been reported that NRF2 can inhibit fatty acid synthesis and promote fatty acid β-oxidation [14]. Our results showed that there was no difference in the expression of key glycolysis enzymes in the siKEAP1 and siNC cells (Fig. 6g), but the expression levels of genes that catalyze fatty acid β-oxidation, the tricarboxylic acid cycle (CS and IDHA1), and the mitochondrial respiratory complex subunits (complex II (succinate-coenzyme Q reductase, SDHB), complex III (coenzyme Q-cytochrome c reductase, Cyt-C), complex IV (cytochrome c oxidase, COX5B), and complex V (ATP synthase, ATP5A)) were significantly increased in siKEAP1 (Fig. 6h–j).

### Bioinformatics analysis of the possible downstream target genes of NRF2

To analyze the target genes that NRF2 directly regulates, the Cistrome Data Browser and GTRD databases were used to figure out the genes related to energy metabolism and myocardium maturation among the downstream genes targeted by NRF2. As shown in Tables 1 and 2, the results showed that NRF2 may directly target genes related to fatty acid β-oxidation metabolism (CPT1A and CPT2), mitochondrial respiratory (IDH, IDH2 complex I (NADHFS, NDUFA2, NDUFA5, NDUFA10, NDUFB2), complex II (SDHB), complex IV (COX6A1, COX5A), complex V (ATP5F1B, ATP5B, ATP5FA)), potassium ion channel genes (KCNC3, KCNA7, KCND3, KCNT1, KCNE4), and cardiac hypertrophy (EDN1).

**Table 1:** Genes related to energy metabolism and myocardial maturation that NRF2 may bind to in the Cistrome Data Browser

Gene	Score	Biological source	Publication
KCNC3	0.011	GM06993, GM12872; lymphoblastoid; blood	Chorley et al., Nucleic Acids Res. 2012
KCNA7	0.001	GM06993, GM12872; lymphoblastoid; blood	Chorley et al., Nucleic Acids Res. 2012
KCND3	0.997	GM11992, GM12763; lymphoblastoid; blood	Chorley et al., Nucleic Acids Res. 2012
CPT1A	2.006	HepG2; hepatocellular carcinoma	ENCODE Project Consortium et al., Nature 2012
NDUFS4	0.936	JHU-06; endothelial cell	Hogan et al., Elife
COX6A1	2.105	LoVo; colon	Yan et al., Cell 2013
ATP5F1B	0.73	GM07000, GM11882; lymphoblastoid; blood	Chorley et al., Nucleic Acids Res. 2012

**Table 2:** Genes related to energy metabolism and myocardial maturation that may bind NRF2 as obtained from the GTRD database

ID	Gene symbol	Ensembl ID	SiteCount
ENSG00000138413	IDH1	ENST00000446179	1
ENSG00000182054	IDH2	ENST00000560061	1
ENSG00000157184	CPT2	ENST00000468572	1
ENSG00000131495	NDUFA2	ENST00000252102	1
ENSG00000128609	NDUFA5	ENST00000466896	1
ENSG00000130414	NDUFA10	ENST00000252711	2
ENSG00000090266	NDUFB2	ENST00000247866	1
ENSG00000073578	SDHA	ENST00000264932	1
ENSG00000178741	COX5A	ENST00000562233	1
ENSG00000110955	ATP5B	ENST00000548647	1
ENSG00000116459	ATP5F1	ENST00000464154	1
ENSG00000078401	EDN1	ENST00000379375	1
ENSG00000107147	KCNT1	ENST00000371757	1
ENSG00000152049	KCNE4	ENST00000488477	1

## Discussion

hiPSC-CMs show spontaneous beating, but their metabolism and functional structure are most similar to those of immature cardiomyocytes in the embryonic period, which limits the further application of hiPSC-CMs in drug screening, disease modeling, and alternative treatment. Currently, most efforts to improve the maturation of hiPSC-CMs focused on biophysical, biochemical, and bioelectrical stimulation with genetic, chemical, and biomechanical approaches, and the study of energy substrates and metabolic pathways for stem cell differentiation of cardiomyocytes is limited [28–30]. It has been shown that the maturation of mitochondrial morphology and function is crucial for the development of hiPSC-CMs [31, 32]. Supplementing free fatty acids (FA) to the medium was a classic way to promote maturation of hiPSC-CMs, by which induced maturation of hiPSC-CMs was even used as a positive control for HIF-1α promoted maturation of hiPSC-CMs [8, 33]. A certain study reported that the expression level of NRF2 was significantly increased in hiPSC-CMs supplemented with FA, indicating the importance of NRF2 in the maturation of hiPSC-CMs [10].

As a transcription factor, NRF2 is related to the expression of more than 500 genes and engages enzymes that regulate mitochondrial respiration [34–36]. The significance of NRF2 in intermediary metabolism is seizing the interest of researchers. In various cellular contexts, NRF2 control multiple mitochondrial functions, most notably the oxidative phosphorylation metabolism by binding to promoter regions of nuclear genes encoding subunits of the five respiratory complexes of the OXPHOS system, thus enhancing OXPHOS respiration and elevates intracellular ATP concentrations [9, 35]. The increased mitochondrial respiratory function is essential for the maturation of hiPSC-CMs. Consistently, after activation of NRF2, we found that the expression of mitochondrial respiratory subunits in hiPSC-CMs was significantly increased, accompanied by enhanced mitochondrial respiration and mitochondrial membrane potential. Out of expectation, the ATP production is not affected by the knock-down of NRF2, we assume that there is a compensatory mechanism involved in this process to meet the energy required for spontaneous beating, but further investigation is needed. NRF2 is also critical in the biogenesis of mitochondria, represented by increasing of the mitochondrial DNA (mtDNA) copy number, which coincides with our result [37, 38]. Mitsuishi et al. found that NRF2 status affects the genes encoding enzymes related with pentose phosphate pathway, which might be the reason why the number of multinucleated cells increased significantly after the activation of NRF2 we detected [39]. By analyzing and detecting the structure and function of cells and mitochondria in hiPSC-CMs, we preliminarily proved that NRF2 plays an important role in promoting the maturation of hiPSC-CMs. However, as only one single hiPSC cell line has been investigated in the present study, it remains to be elucidated whether NRF2 will play a similar role in other hiPSC cell lines of different origin. Further evaluation on more hiPSC cell lines from diverse source to conform the effect of NRF2 is currently on the schedule in our group.

On the other hand, in regenerative medicine and alternative therapy, transplanted hiPSC-CMs die quickly due to severe inflammation at the site of myocardial injury. ROS is considered to be a main cause of cell death. NRF2 inducers were found to exhibit a protective effect in cardiac remodeling. Cell culture models of cardiomyocytes have revealed the cytoprotective effect of NRF2 via reduction of ROS and inducing the production antioxidant and detoxification enzymes. Coincidently, the activation of NRF2 with resveratrol or curcumin showed a protective effect against inflammation and endothelial cell dysfunction [15, 40]. Therefore, we speculated that enhanced NRF2 expression in hiPSC-CMs might also reduce the inflammatory response at the injured site. Bioinformatics analysis also revealed that NRF2 may regulate genes such as ion channels in addition to mitochondrial respiration. Accordingly, we hypothesize that NRF2 not only promotes cell maturation by changing mitochondrial respiration, but it may also combine multiple pathways to promote hiPSC-CMs maturation. Based on the above, the mechanism of NRF2 will be further screened and verified through ChIP-seq.

## Conclusions

This study demonstrated that NRF2 is indispensable for the maturation of hiPSC-CMs, and the activation of NRF2 can be used as a key technical means to enhance the maturity of hiPSC-CMs. This improvement in understanding the mechanism of hiPSC-CMs maturity promotes potential applications of hiPSC-CMs in cardiac drug screening, disease modeling, and alternative treatments.

## Supplementary Information

**Additional file 1: Figure S1.** Characterization of hiPSCs. (A) Cardiomyogenic differentiation protocol including hiPSC expansion, cardiomyocyte differentiation, purification, culture, and treatments. The different media and study factors used on different days of the timeline are indicated. (B) Immunostaining for SOX2 and Nanog in the hiPSCs. qPCR of OCT4 (C) and Nanog (D) during differentiation. The abscissa represents the number of days of differentiation. *n* = 3; the means ± SEM are shown. ****P < 0.0001. **Figure S2**. siRNA knockdown of NRF2 decreased hiPSC-CMs functional maturation. hiPSC-CMs were analyzed by calcium transient kinetics evaluated with Fluo-4 AM. *n* > 3 cells per condition, three biological replicates. (A) Representative calcium transient; (B) calcium transient amplitude (F/F0). (C) Maximum calcium transient upstroke velocities. (D) Maximum calcium transient decay velocities. The means ± SEM are shown. ** P < 0. 01, **** P < 0. 0001. **Figure S3.** siRNA knockdown of NRF2 decreased hiPSC-CMs mitochondrial maturation. (A) mtDNA copy numbers of siNRF2- and siNC-hiPSC-CMs were determined by qPCR; *n* = 3. (B) Mitochondrial staining by MitoTracker Red (red, mitochondria; blue, nucleus); Scale bar = 10 μm. (C-D) Mitochondrial membrane potential (mtΔΨ) of siNRF2 and siNC hiPSC-CMs are determined by JC-1 staining. (C) Quantitative analysis of the mitochondrial membrane potential (mtΔΨ); *n* > 4 cells per condition, three biological replicates. (D) Representative mtΔΨ in of siNRF2 and siNC hiPSC-CMs. Scale bar = 10 μm. The means ± SEM are shown. * P < 0. 05, **** P < 0. 0001. **Figure S4.** Cell proliferation of siKEAP1 and siNC hiPSC-CMs as analyzed by an BeyoClick™ EdU cell proliferation kit with Alexa Fluor 488 and immunostaining of cTnT (red). **Figure S5.** NRF2 promotes the mitochondrial maturation of hiPSC-CMs. (A) mtDNA copy numbers of siKEAP1 and siNC hiPSC-CMs were determined by qPCR. n = 3. (B) Mitochondrial staining by MitoTracker Red (red, mitochondria; blue, nucleus); Scale bar = 10 μm. (C, E) Mitochondrial membrane potential (mtΔΨ) of the siKEAP1 and siNC hiPSC-CMs are determined by JC-1 staining. (C) Quantitative analysis of the mitochondrial membrane potential (mtΔΨ). *n* > 8 cells per condition, three biological replicates. (E) Representative mtΔΨ in of the siKEAP1 and siNC hiPSC-CMs; Scale bar = 10 μm. (D) ATP content of the siKEAP1 and siNC hiPSC-CMs were determined using an ATP assay kit; n = 3. The means ± SEM are shown. * P < 0. 05, *** P < 0. 001. **Online Table 1**. List of primers used for Q-PCR and mtDNA copy number.

## Data Availability

The datasets used and/or analyzed during the current study are available from the corresponding author on reasonable request.

## Abbreviations

ACAA2: Acetyl-CoA acyltransferase 2
ATP5A, ATP5B, ATP5FA, ATP5F1B: ATP synthase (complex V)
CACNA1C: Calcium ion channel
COX4, COX5A, COX5B, COX6A1: Cytochrome C oxidase subunits (complex IV)
CPT1A and CPT2: Carnitine palmitoyl transferase
CS: Citrate synthase
cTnT: Cardiac troponin
CX43: Connexin43
Cyt-C: Coenzyme Q-cytochrome c reductase (complex III)
ECH1: Enoyl-CoA hydratase 1
EDN1: Endothelin 1
FA: Fatty acid
FAO: Fatty acid oxidation
FCCP: Carbonyl cyanide-p-trifluoromethoxyphenylhydrazone
GAPDH: Glyceraldehyde 3-phosphate dehydrogenase
GSK3-β: Glycogen synthase kinase 3-β
hiPSC-CMs: Human induced pluripotent stem cell-derived cardiomyocytes
HK2: Hexokinase
IDH, IDH2, IDHA1: Isocitrate dehydrogenase
KCNA7, KCNC3, KCND3, KCNE1, KCNE4, KCNJ2, KCNT1: Potassium ion channel
KEAP1: Kelch-like ECH-related protein 1
MPC1: Mitochondrial pyruvate carrier 1
MYH6, MYH7: Myosin heavy chain
MYL7: Myosin Light Chain
NDUFA1, NADHFS, NDUFA2, NDUFA5, NDUFA10, NDUFB2: NADH-coenzyme Q reductase (complex I)
NRF2: Nuclear factor erythroid 2 p45-related factor 2
OCR: Oxygen consumption rate
OGDH: Oxoglutarate dehydrogenase
PDHA1: Pyruvate dehydrogenase E1 subunit alpha 1
PFK: Phosphofructokinase
PGM1: Pluripotency growth master 1
PKM: Pyruvate kinase muscle
Q-PCR: Quantitative real-time polymerase chain reaction
SCN5A: Sodium ion channel
SDHB: (Complex II) succinate-coenzyme Q reductase
SOX2: SRY-Box transcription factor 2
TNNT2: Troponin T2
