# Supplementary material for: NRF2 is required for structural and metabolic maturation of human induced pluripotent stem cell-derived ardiomyocytes
Source: Stem Cell Res Ther. 2021 Mar 24;12:208. doi: 10.1186/s13287-021-02264-2 (PMC7992990; doi:10.1186/s13287-021-02264-2)
Supplement: Supplementary file 2 — Additional file 1: Figure S1. Characterization of hiPSCs. (A) Cardiomyogenic differentiation protocol including hiPSC expansion, cardiomyocyte differentiation, purification, culture, and treatments. The different media and study factors used on different days of the timeline are indicated. (B) Immunostaining for SOX2 and Nanog in the hiPSCs. qPCR of OCT4 (C) and Nanog (D) during differentiation. The abscissa represents the number of days of differentiation. n = 3; the means ± SEM are shown. ****P < 0.0001. Figure S2. siRNA knockdown of NRF2 decreased hiPSC-CMs functional maturation. hiPSC-CMs were analyzed by calcium transient kinetics evaluated with Fluo-4 AM. n > 3 cells per condition, three biological replicates. (A) Representative calcium transient; (B) calcium transient amplitude (F/F0). (C) Maximum calcium transient upstroke velocities. (D) Maximum calcium transient decay velocities. The means ± SEM are shown. ** P < 0. 01, **** P < 0. 0001. Figure S3. siRNA knockdown of NRF2 decreased hiPSC-CMs mitochondrial maturation. (A) mtDNA copy numbers of siNRF2- and siNC-hiPSC-CMs were determined by qPCR; n = 3. (B) Mitochondrial staining by MitoTracker Red (red, mitochondria; blue, nucleus); Scale bar = 10 μm. (C-D) Mitochondrial membrane potential (mtΔΨ) of siNRF2 and siNC hiPSC-CMs are determined by JC-1 staining. (C) Quantitative analysis of the mitochondrial membrane potential (mtΔΨ); n > 4 cells per condition, three biological replicates. (D) Representative mtΔΨ in of siNRF2 and siNC hiPSC-CMs. Scale bar = 10 μm. The means ± SEM are shown. * P < 0. 05, **** P < 0. 0001. Figure S4. Cell proliferation of siKEAP1 and siNC hiPSC-CMs as analyzed by an BeyoClick™ EdU cell proliferation kit with Alexa Fluor 488 and immunostaining of cTnT (red). Figure S5. NRF2 promotes the mitochondrial maturation of hiPSC-CMs. (A) mtDNA copy numbers of siKEAP1 and siNC hiPSC-CMs were determined by qPCR. n = 3. (B) Mitochondrial staining by MitoTracker Red (red, mitochondria; blue, nuc [file 13287_2021_2264_MOESM1_ESM.doc]

**NRF2 is required for structural and metabolic maturation of human induced pluripotent stem cell-derived cardiomyocytes**

Xinyuan Zhang^1,4^, Liang Ye^1,4^, Hao Xu^2,4^, Qin Zhou^1,4^, Bin Tan^1,4^, Qin Yi^1,4^, Liang Yan^1,4^, Min Xie^1,4^, Yin Zhang^1,4^, Jie Tian^3,4^, Jing Zhu^1,4^ *

1. Department of Pediatric Research Institute, Ministry of Education Key Laboratory of Child Development and Disorders; National Clinical Research Center for Child Health and Disorders (Chongqing); China International Science and Technology Cooperation base of Child development and Critical Disorders; Children's Hospital of Chongqing Medical University, Chongqing, P.R China;

2. Department of Clinical Laboratory; Ministry of Education Key Laboratory of Child Development and Disorders; National Clinical Research Center for Child Health and Disorders (Chongqing); China International Science and Technology Cooperation base of Child development and Critical Disorders; Children's Hospital of Chongqing Medical University, Chongqing, P.R China;

3. Department of Cardiovascular (Internal Medicine), Ministry of Education Key Laboratory of Child Development and Disorders; National Clinical Research Center for Child Health and Disorders (Chongqing); China International Science and Technology Cooperation base of Child development and Critical Disorders; Children's Hospital of Chongqing Medical University, Chongqing, P.R China;

4. Chongqing Key Laboratory of Pediatrics, Chongqing, China

* Corresponding author: Jing Zhu, Children's Hospital of Chongqing Medical University, Box 136, No. 3 Zhongshan RD, Yuzhong district, Chongqing, 400014, China

Email: jingzhu@cqmu.edu.cn


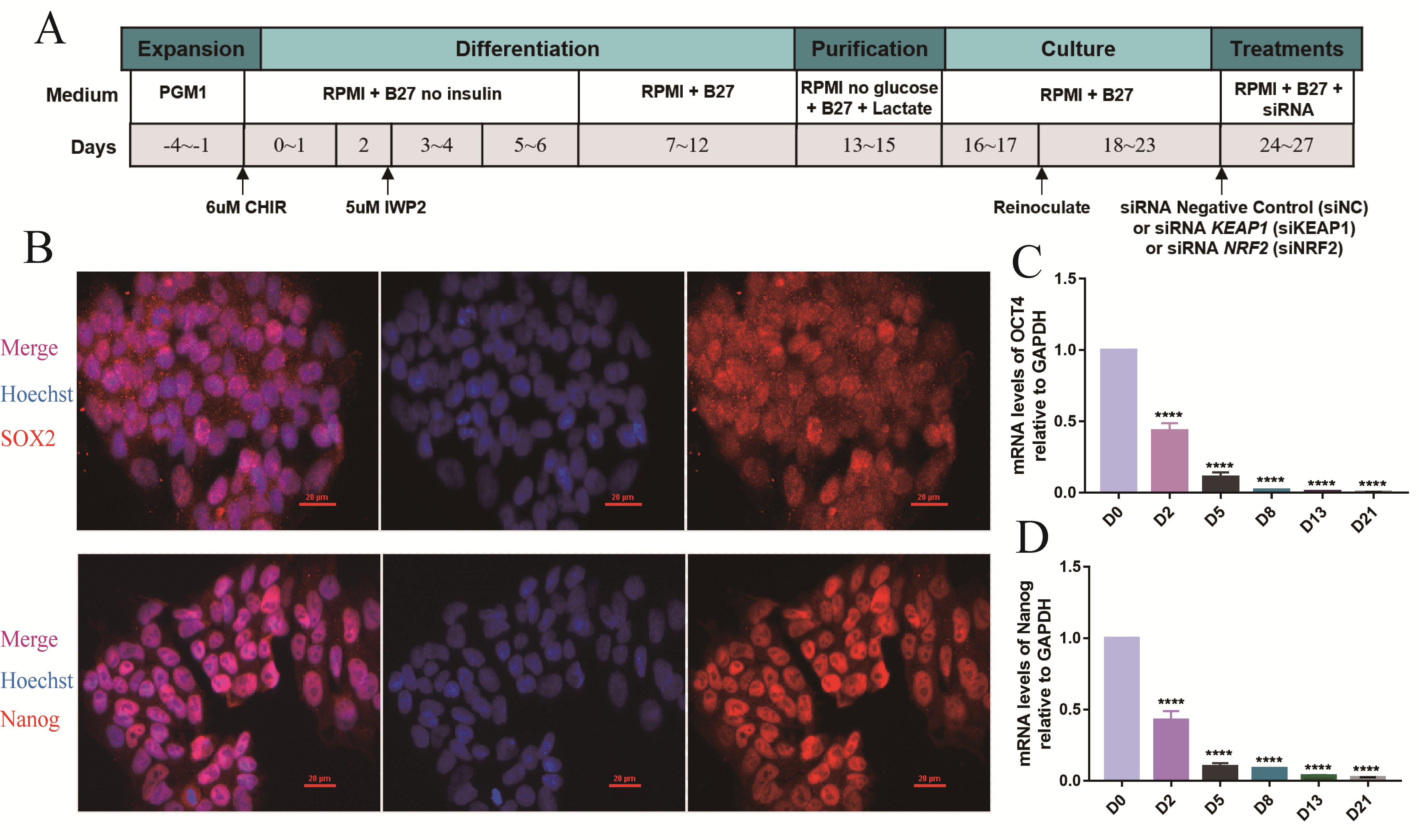


Figure S1. Characterization of hiPSCs. (A) Cardiomyogenic differentiation protocol including hiPSC expansion, cardiomyocyte differentiation, purification, culture, and treatments. The different media and study factors used on different days of the timeline are indicated. (B) Immunostaining for SOX2 and Nanog in the hiPSCs. qPCR of cardiac marker genes OCT4 (C) and Nanog (D) during differentiation. The abscissa represents the number of days of differentiation. n = 3; the means ± SEM are shown. ****P ˂ 0.0001.


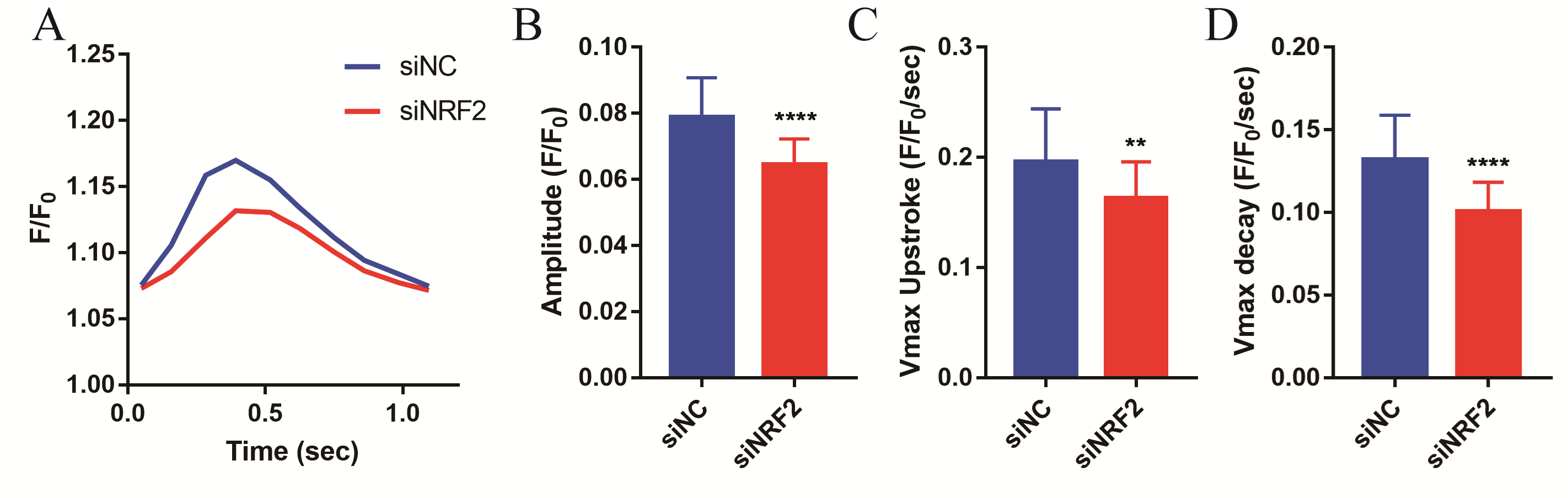


Figure S2. siRNA knockdown of NRF2 decreased hiPSC-CM functional maturation. hiPSC-CMs were analyzed by calcium transient kinetics evaluated with Fluo-4 AM. n > 3 cells per condition, three biological replicates. (A) Representative calcium transient; (B) calcium transient amplitude (F/F0). (C) Maximum calcium transient upstroke velocities. (D) Maximum calcium transient decay velocities. The means ± SEM are shown. ** P ˂ 0. 01, **** P ˂ 0. 0001.


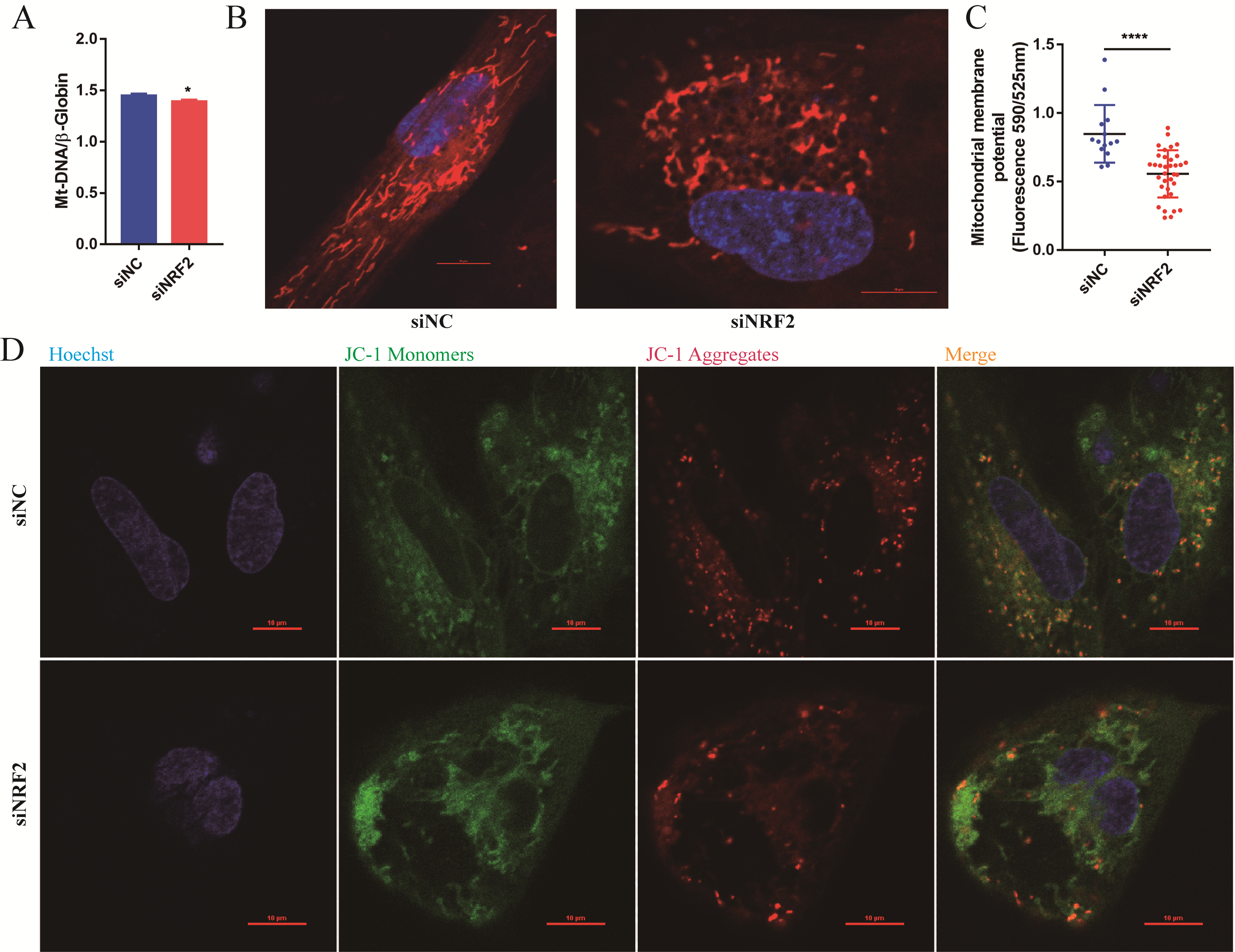


Figure S3. siRNA knockdown of NRF2 decreased hiPSC-CM mitochondrial maturation. (A) mtDNA copy numbers of siNRF2- and siNC-hiPSC-CMs were determined by qPCR; n = 3. (B) Mitochondrial staining by MitoTracker Red (red, mitochondria; blue, nucleus); Scale bar = 10 μm. (C-D) Mitochondrial membrane potential (mtΔΨ) of siNRF2 and siNC hiPSC-CMs are determined by JC-1 staining. (C) Quantitative analysis of the mitochondrial membrane potential (mtΔΨ); n > 4 cells per condition, three biological replicates. (D) Representative mtΔΨ in of siNRF2 and siNC hiPSC-CMs. Scale bar = 10 μm. The means ± SEM are shown. * P ˂ 0. 05, **** P ˂ 0. 0001.


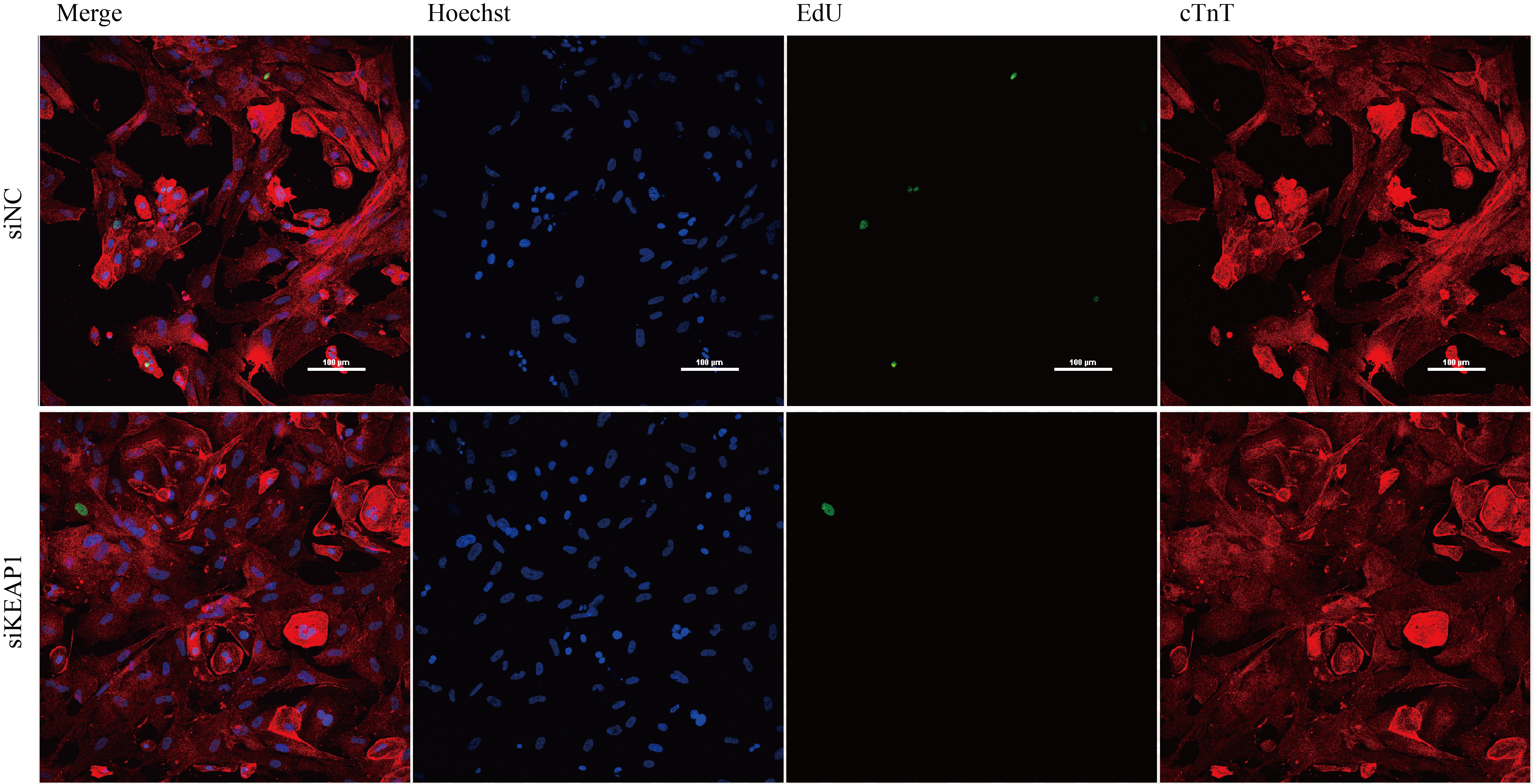


Figure S4. Cell proliferation of siKEAP1 and siNC hiPSC-CMs as analyzed by an BeyoClick™ EdU cell proliferation kit with Alexa Fluor 488 and immunostaining of cTnT (red).


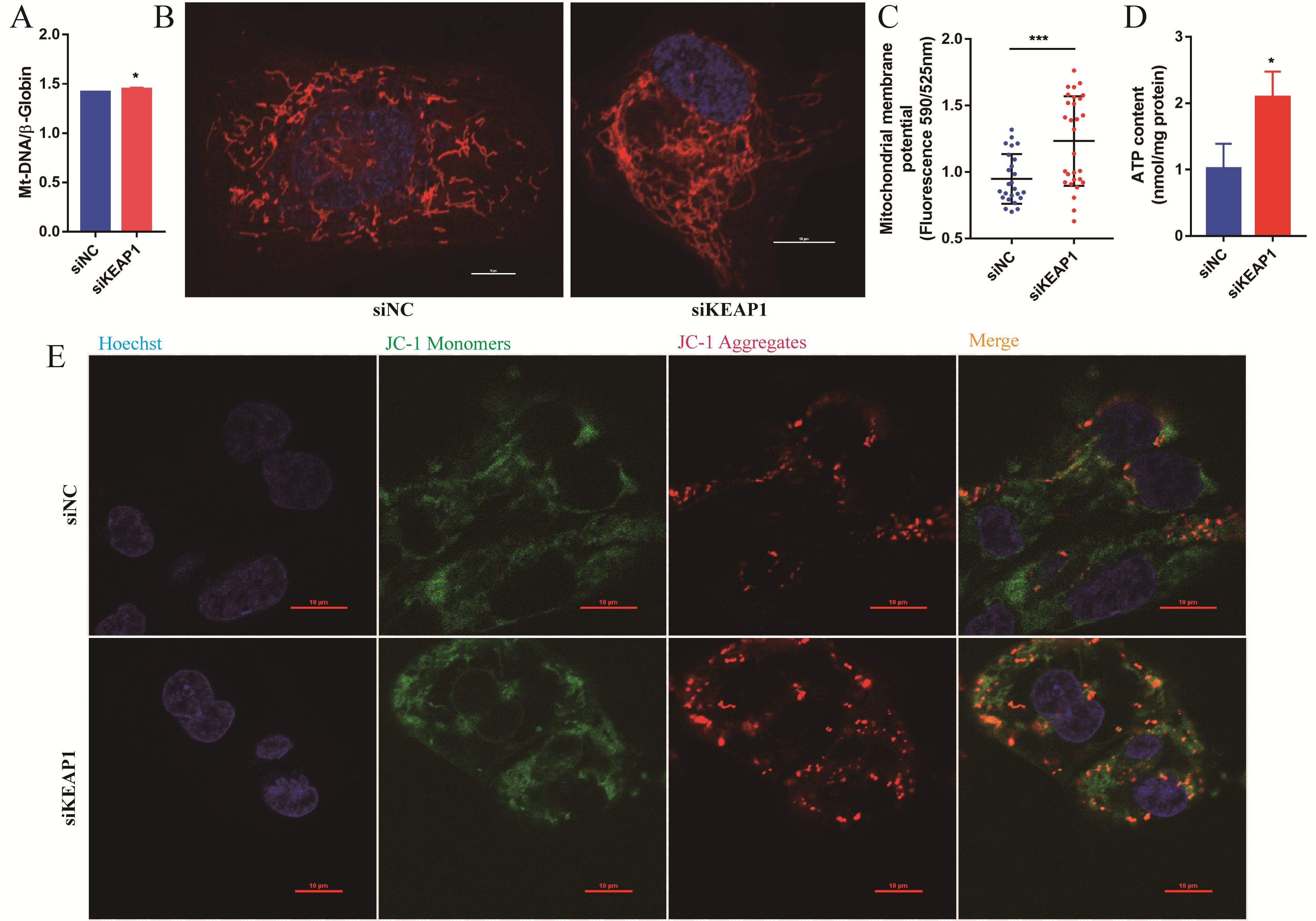


Figure S5. NRF2 promotes the mitochondrial maturation of hiPSC-CMs. (A) mtDNA copy numbers of siKEAP1 and siNC hiPSC-CMs were determined by qPCR. n = 3. (B) Mitochondrial staining by MitoTracker Red (red, mitochondria; blue, nucleus); Scale bar = 10 μm. (C, E) Mitochondrial membrane potential (mtΔΨ) of the siKEAP1 and siNC hiPSC-CMs are determined by JC-1 staining. (C) Quantitative analysis of the mitochondrial membrane potential (mtΔΨ). n > 8 cells per condition, three biological replicates. (E) Representative mtΔΨ in of the siKEAP1 and siNC hiPSC-CMs; Scale bar = 10 μm. (D) ATP content of the siKEAP1 and siNC hiPSC-CMs were determined using an ATP assay kit; n = 3. The means ± SEM are shown. * P ˂ 0. 05, *** P ˂ 0. 001.

Online Table 1. List of primers used for Q-PCR and mtDNA copy number.

| ACAA2 | GTCTGCTGGCAAAGTCTCACCTG | GATTCCCACACGCAAACCAACATG |
| --- | --- | --- |
| ATP5A | CCCAGTTCGGTTCTGACCTC | TCCCCTTACACCCGCATAGA |
| CACNA1C | AGTCTCCACCCGCCACCAAG | GCTCCTCCTCATCCTCTTCTCCTG |
| COX5B | TGTGAAGAGGACAATACCAGC | CCAGCTTGTAATGGGCTCC |
| CPT1A | GTGAGCGACTGGTGGGAGGAG | TGCTGCCTGAATGTGAGTTGGAAG |
| CS | GACTTTCAGGCAGCAACATGG | ACTAAAGCCTCGGAAACGGA |
| Cyt-C | TCCTTGGACTTAGAGAGTGGGG | CTTGTGCTTGCCTCCCTTTTC |
| ECH1 | GCCACGACTGAGAACAAGGAACTG | AGAAACAACTGTCATCGCCCATCC |
| GAPDH | GAAATCCCATCACCATCTTCCAG | AAATGAGCCCCAGCCTTCTC |
| HK2 | AAGCCCTTTCTCCATCTCCT | CTTCTTCACGGAGCTCAACC |
| IDH1 | CAACTCTTCGCCAGCATATCA | CATCTCTACCACAGAACCGCC |
| KCND3 | TGGGCAGCACGGAGAAGGAG | TTGAGCACGCAGCGGAACAC |
| KCNE1 | ACCACCAAACAGACAAGCCCTTC | TCTCTTCCTCCTGAGCACGGTTC |
| KCNJ2 | GCCTCCACACTTGCTCTTCTGATC | CACCTCCCTGTTTCCCTGCTTTG |
| KEAP1 | TGAGCCAGAGCGGGATGAGTG | CCCACGGCATAAAGGAGACGATTG |
| MPC1 | CCACTCGGGGTCTGGTGT | CAGCTTCCCAAGGTCCCTAC |
| MYH6 | GTTCAAGAAGATAGTGGAACGC | GGCTTGATCTTGAAGTAGAGCT |
| MYH7 | GCTTCGGGAAATTCATTCGAAT | TCAGGCTTTTTGTTAGACAGGA |
| MYL7 | CGTCTTCCTCACGCTCTTTG | GGGTGTCAGGGCGAACAT |
| NANOG | ATACCTCAGCCTCCAGCAGA | TCTGCGTCACACCATTGCTA |
| NDUFA2 | TCCCGACCTACCCATCCTAAT | CCAAATGCTGAAGAGAGAGAGG |
| NRF2 | ACGGTATGCAACAGGACATTGAGC | TTGGCTTCTGGACTTGGAACCATG |
| OCT4 | AGTGCCCGAAACCCACA | GAGACCCAGCAGCCTCAAA |
| OGDH | TTGGCTGGAAAACCCCAAAAG | TGTGCTTCTACCAGGGACTGT |
| PDHA1 | CGGGCTCACGGCTTTACTTT | GAATATCTGGCCCTGCGCTC |
| PFK1 | GAGCGGTTTTTACAGAGTGGA | GGACAAAGAAGACACGGGCA |
| PKM | GTCTGCTCACCAGGTGGCC | AACATTCATGGCAAAGTTCACCCG |
| SCN5B | TAGAGGAGCGGAAGACCATCAAGG | GCAGCATCTCCAGCACGAAGAC |
| SDHB | CTCAGGAAGGCAAGCAGCAGTATC | ATTTGTCTCCGTTCCACCAGTAGC |
| TNNT2 | TTCACCAAAGATCTGCTCCTCGCT | TTATTACTGGTGTGGAGTGGGTGTGG |
| mt-ND1 | GGCTACATACAATTACGCAAAG | TAGAATGGAGTAGACCGAAAGG |
| β-globin | TGTGTCAGAAGCAAATGTAAGCAA | AAGAGCCAAGGACAGGTACGG |
